# Controlled release of pharmaceutical agents using eutectic modified gelatin

**DOI:** 10.1007/s13346-021-00998-3

**Published:** 2021-05-08

**Authors:** Wanwan Qu, Idrees B. Qader, Andrew P. Abbott

**Affiliations:** 1 School of Chemistry, University of Leicester, Leicester, LE1 7RH UK; 2 Pharmaceutical Chemistry Department, College of Pharmacy, Hawler Medical University, Erbil, Kurdistan Region Iraq

**Keywords:** Dissolution rate, Solubility, Deep eutectic solvents, And drug delivery

## Abstract

Deep eutectic solvent (DES) is a class of ionic liquids, consisting of a mixture generally formed by combining hydrogen bond donors (HBDs) such as alcohols, amides and carboxylic acids with various quaternary ammonium salts. The decrease in melting points of the constituents is due to the charge delocalization during formation of hydrogen bonding between the hydrogen bond acceptor with the hydrogen bond donor. This can be considered one of the main reasons for increasing solubility and absorption of DESs. Most active pharmaceutical ingredients (APIs) have polar functional groups containing amide, carboxylic acid, alcohol or quaternary ammonium groups. These tend to increase the melting point of the compounds, but they can be used to form eutectic mixtures. While this concept has previously used, the combination of quaternary ammonium salts with amides, carboxylic acids and alcohols can result in large depressions of freezing points and so-called deep eutectic solvents are formed. DESs mix readily with water and so could increase the uptake of APIs. In this study, pharmaceutical deep eutectic solvents (PDESs) are formulated from 3 APIs: imipramine HCl, ascorbic acid and catechol. These PDESs were used to plasticise gelatine. It is shown that the materials formed can be used to increase the rate of API uptake via both oral and transdermal delivery modes. Thus, the concentration of the PDESs in solution reaches the maximum before the pure drugs. Particularly for catechol, after 1 s, the dissolution of the PDESs was more than twice that of the pure drug. Moreover, the transdermal delivery mode uptake of the PDES based on imipramine HCl from the patch after 15 min was found to be 65% compared with just imipramine HCl which released only 20%.

**Graphical abstract:**

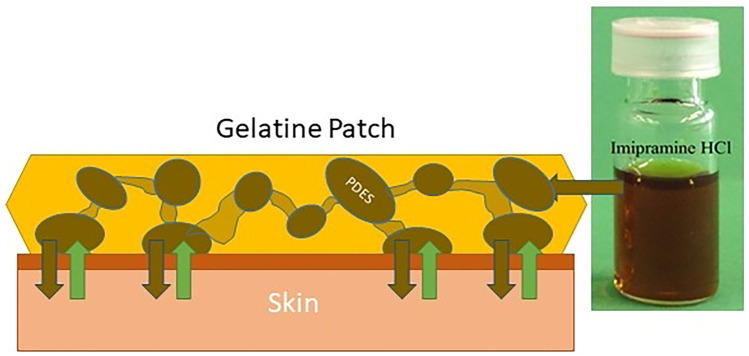

**Supplementary information:**

The online version contains supplementary material available at 10.1007/s13346-021-00998-3.

## Introduction

Oral administration, being more natural and less invasive than other conventional methods such as intramuscular injection, is seen as the preferred mode of most drug administration. However, the physiological availability of many drugs administered via this route is poor for several reasons, including a lack of stability in the gastrointestinal environment, resulting in degradation of the active compound prior to its absorption. In order to circumvent this problem, many polymers have been used to modulate drug delivery including localised drug delivery using transdermal patches. Gelatin is a common water-soluble functional biopolymer which has been widely used for pharmaceutical use. The polymer used needs to be fairly dry, powdered and have uniform particle size to maintain even density to ensure even drug or active pharmaceutical ingredient (API) delivery.

Pharmaceutical treatment needs to ensure a known and constant dose of API, and so solubility and activity differences arising from issues such as polymorphs and functional groups need to be controlled. Eutectic mixture commonly consists of two or more components which has a lower melting point than its individual components. Thus, by decreasing the melting point, the solubility and efficiency of drug delivery increase [1]. For example, a eutectic composition of the local anaesthetic drugs lidocaine and prilocaine has been used to enhance the transdermal permeation of lidocaine from a cream formulation [2]. Of particular interest are deep eutectic solvents, DESs, which are mixtures of quaternary ammonium salts and hydrogen bond donors [3, 4]. The term ionic liquid is different from DES because ionic liquids includes only ions (cation and anions) which interact through electrostatic forces. However, DESs include both ions and neutral molecules combined through hydrogen bonds with the anion of a salt [5].

DESs provide a novel method by which liquids can be formulated from two solids by forming a eutectic which melts at or below ambient temperature. We have recently shown that this approach can be used to liquefy APIs, to form pharmaceutical deep eutectic solvents PDESs. Given that many APIs contain –COOH, –OH or –CONH_2_ functional groups or amine groups which can be quaternised using HCl, there are a wide range of these PDESs which can be formulated. This approach could overcome solubility issues in water as the eutectics prevent recrystallization of the active ingredient when dispersed in water. Polymorphic APIs such as adiphenine and ranitidine can form PDESs with a range of hydrogen bond donors which can also be a pharmaceutical active ingredient e.g. aspirin [6].

In addition, the same principle has been used to develop pharmaceutical ionic liquids (PILs). For example, propantheline bromide and acesulfamate were formulated into PILs, and the latter is an artificial and pharmaceutical additive sweetener [7]. This formulation method is in its infancy; thus, biological and toxicological testing are necessary to fully understand how these PILs act inside the body [6]. There is an ever-increasing demand for novel medicine delivery systems in an endeavour to increase drug solubility and thereby bioavailability, to limit toxicity and improve pharmacokinetics [8]. Of these, transdermal creams and patches are one of the most common [9]. A variety of synthetic and biopolymers have been studied with drugs held in reservoirs, solid matrices, hydrogels and non-hydrogels. A common approach is the use of gelatin hydrogels as they resemble living tissues closely in their physical properties because of their relatively high moisture content and soft and rubbery consistency. Many approaches have been taken to increase the efficiency of transdermal delivery systems such as the use of skin enhancers [10]. One issue is the ability of hydrogels to lose water, and it is this issue that this paper attempts to address.

Previously, we have shown that ILs can be used to modify polymer materials [11], starch and proteins like zein by breaking the hydrogen bonding networks [12]. In the current study, gelatin in modified with PDESs and the mechanical properties of the materials are characterised. We demonstrate the application of PDESs for API delivery from modified gelatin films through both an oral and transdermal route. For the latter method, skin mimics are made using brine soaked bovine leather and porcine cut demonstrating the different rates of drug delivery depending on fat content.

## Methods

All materials employed in this study were used as received and their sources, and purities are listed in Table 1.

**Table 1:** Sources and purity (by mass) of chemicals used in this study

Chemicals	Source	Purity %
Glycerol	Fischer	98
Choline chloride	Sigma-Aldrich	≥ 98
Aspirin	Sigma-Aldrich	≥ 99
Catechol	Sigma-Aldrich	≥ 99
Ascorbic acid	Sigma-Aldrich	99
Imipramine HCl	Sigma-Aldrich	≥ 99
Gelatin	Sigma-Aldrich	CAS 9000-70-8

The protein powder used in this study was fish skin gelatin; DES components include glycerol, choline chloride (ChCl), imipramine HCl, ascorbic acid, aspirin and catechol. The four PDESs were catechol: ChCl (1:1), aspirin: ChCl (1:1), imipramine HCl: glycerol (1:2) and ascorbic acid: ChCl (1:2). The method of synthesis was the same for all of the liquids. After mixing, the liquid was then put into a Thermo Scientific Heraeus oven (50 C, overnight). To finish off the melting process, the partially melted mixture was placed on a hotplate stirrer at approximately 90 °C until a clear homogenous liquid was formed for each.

While numerous PDES formulations have been shown to be liquids at ambient temperature, the four listed above were chosen as they are active in the UV-vis portion of the spectrum enabling spectrophotometric assays of solubility. This is a rapid, accurate and sensitive method for the determination of API in pharmaceutical dosage forms and has been used by many researchers [13]. The absorption spectra of aspirin, ascorbic acid, catechol and imipramine hydrochloride exhibit absorption maxima at about 276 nm, 244 nm, 277 nm and 251 nm, respectively.

Initial tests revealed that the absorption peaks of all drugs remained the same for the pure API, and the PDES, except for aspirin, which was suspected to decompose through hydrolysis, yielding salicylic and acetic acids, which corresponds to the observed peak shift from 277 nm (aspirin) to 303 nm (salicylic acid). This is included as a warning check (which was not included in the initial publication) that chemical reaction between the two components should be checked because hydrolysis and esterification reactions can occur with acidic components. The spectra for the formulations with catechol, imipramine HCl and ascorbic acid remained the same as the starting material indicating no significant reaction between the components took place. Hence, three drugs were chosen for further tests, and the prepared PDESs from these drugs are liquid at room temperature.

**Fig. 1:**
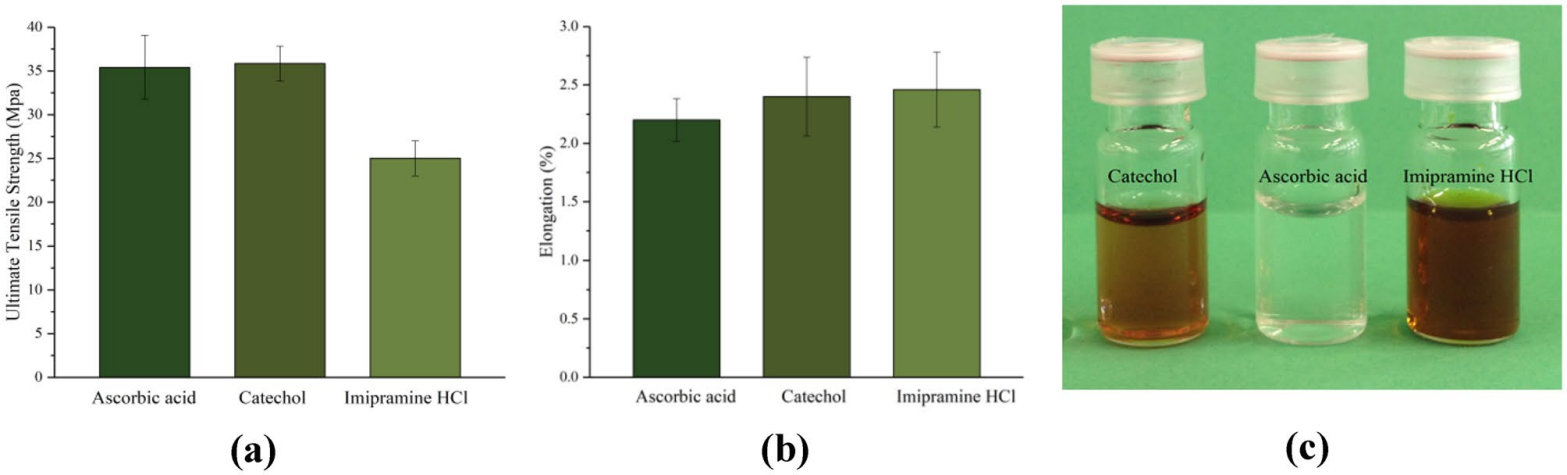
**a** Ultimate tensile strength and **b** elongation for gelatin samples with 20% of different PDESs after 12 h of setting time **c** samples of PDES ascorbic acid: ChCl (1:2), catechol: ChCl (1:1) and imipramine HCl: glycerol (1:2). The error bars have been measured for three readings

A set of standard solutions were prepared from pure substances in the dissolving media (0.1 M HCl to approximate the contents of the stomach), and calibration curves were produced as a function of concentration. In all cases, straight line correlations between absorbance and concentration were observed in accordance with the Beer-Lambert law. Noted that the error bars for all plots are within the dimensions of the plot symbol, which shows the high reproducibility of replicate (three times) determinations as reported in Fig. 1S (electronic supplementary information).

In terms of dissolution studies, pharmaceutical ingredient alone and its eutectic mixtures were directly added to 500 ml of 0.1 mol/l HCl solution at 37 °C, respectively. Dissolution studies were performed according to CP (2005 Edition). The rotation speed of the paddle was adjusted to 100 rpm. Dissolution samples were collected every minute and analyzed by direct UV spectrophotometry. The cumulative amount of drug released was calculated and plotted versus time.

Furthermore, to make tablets, the DES was mixed with gelatin (25 wt% DES + 75 wt% gelatin), and the powder was pressed in a mould at 75 °C at a force of 120 kN for 10 min. At the end of this time, the tablets were removed from the mould and dissolved in 0.1 mol dm^−3^ HCl. Aliquots of the HCl solution were taken as a function of time, and the PDES concentration in solution was determined using UV-Vis spectroscopy using the calibration curves shown in Fig. 1S.

Regarding the transdermal delivery, the leather sample was prepared by soaking a chromium tanned bovine leather sample in saline solution for 30 min to achieve a similar moisture and electrolyte level as human skin, which varies between 15 to 30%. The hydration level for the leather samples was measured using a moisture meter (Testo 606-1 Moisture Meter), ten measurements were taken each time and the average hydration levels for the leather before and after soaking were calculated to be 13 and 27%, respectively.

## Result and discussions

### Mechanical properties of PDES modified gelatin

Previous studies modifying gelatin with a ChCl: 2 glycerol, DES, showed that relatively strong materials could be produced [12]. The ultimate tensile strength and ductility were affected significantly by the time. This is related to the fact that components of DES were contacted with each other because of the slow ingress of the DES into the crystalline gelatin structure. Once the materials were compression moulded into a sheet, however, the mechanical properties remained unchanged. Aqueous hydrogels need a high water content to solubilise the API. In the case of PDESs, the liquid content is reduced as the API is typically 30–70% of the liquid formulation enabling either a smaller patch or smaller pill to be used.

The physical properties of DESs are significantly altered by their chemical composition. The viscosity, for example, can be changed by two orders of magnitude with relatively small changes in the structure of the hydrogen bond donor [14]. Accordingly, experiments were carried out comparing the physical properties between gelatin samples prepared using different PDESs. It may be expected that a less viscous DES may result in weaker interactions between polymer chains and hence a weaker material. Contrary to what was expected, Fig. 1 showed no indication that the mechanical properties being influenced by the viscosity of the modifier, in this case PDESs. Taking the standard deviations into consideration, the samples showed similar ultimate tensile strengths and elongations at break, which are indications of the strength and flexibility of the drug patches, respectfully. This implies that the gelatin structure changes caused by plasticization dominate the mechanical behaviour of the liquid infused system rather than the fluidity of the reservoirs. This is a useful as it means that the material properties are not significantly affected by the API used.

**Fig. 2:**
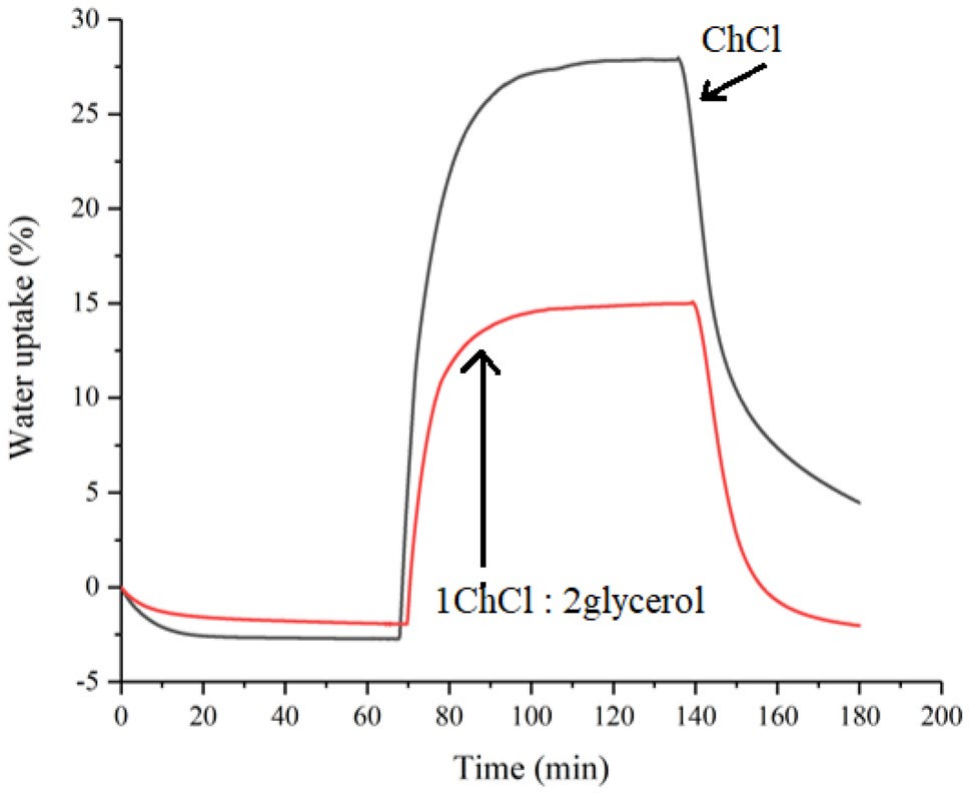
Comparison of water uptake by pure ChCl and a 1ChCl:2 glycerol mixture measured by TGA with the humidity attachment

A useful characteristic of the PDES modified gelatin films is that it can be naturally tacky depending on their method of preparation. This is particularly important when the material is used for a transdermal delivery device. Many methods have been used to test the adhesiveness of a patch material, but in this study, the adhesion was measured using the compression method which is commonly used for the determination of the stickiness of food materials such as pasta. It measures the maximum force required to remove a 1 kg mass after it has been contacted with a surface for a given time (10 s). The stickier the product, the greater the negative force will be exerted on the compression plate. The data below were measured for a 20 wt% ChCl: ascorbic acid in gelatin film. The tackiness was seen to change with the mixing time of the two components prior to pressing. For the sample where the sheet was pressed immediately after mixing, the sample was not tacky and did not stick at all. Increasing the setting time to 6 h produced a sample which required a force of 2.48 N to remove from the compression plate. The material increased its tackiness requiring 31.83 N after 24 h, and 40.96 N after 48 h setting. From the results, it can be concluded that, not only the gelatin patches appeared to have relatively high adhesion, but also, it can be modified by additional setting time, which is important in the sense that the surface property can then be designed by controlling the production procedure, to tailor for different patient needs. Similar properties have been observed by many other researchers and been used in other biomaterial fields; one example is the use of alginate/gelatin sticky gel in the fixations of the implant and implant-bone integration after joint arthroplasty to prevent inflammation and promote bone regeneration [15]. The mechanical properties of these materials are important depending on whether they are used for tablets or patches. The hardness and flexibility data for these materials are detailed in the supplementary information (Fig. 2S).

The transdermal route is an extremely attractive option for delivery; however, it is important that the API should not irritate to the human skin. Irritation can be due to many reasons, one of which is related to the dryness of the skin [16]. Despite that, the stratum corneum, SC, normally experiences low relative humidity, the exposure to very dry environments can still result in defective skin conditions. The continuous hydration of the SC is therefore crucial for maintaining a healthy skin in order to play its role as a water regulator. Choline chloride and quaternary ammonium chlorides, in general, are a major component of PDESs. Apart from its ability of acting as the hydrogen bond donor to form eutectic solvents with many APIs, it has been documented to be used as a humectant in some skin care [17], similar to humectants naturally existing in the SC. More commonly, moisturizers containing glycerol have been used for treatment or prevention of defective dry skin conditions to make the SC softer and more pliable. The biological effects of glycerol are traditionally attributed to its chemical structure. It has three hydrophilic hydroxyl groups, that are responsible for its water solubility and hygroscopicity, which is the ability to attract and hold water molecules from the surrounding environment, usually at normal or room temperature. Pure glycerol can absorb up to its own weight in water [18].

When being used in large amount or undiluted, both choline chloride and glycerol can draw moisture not only from the environment, but from the lower layers of skin due to their relatively high hydrophilicity. When applied topically, this could cause the skin to dry from the inside out, making them potentially irritating to the skin. All salts are categorised as being irritants as they all absorb moisture to some extent. The amount of water which can be absorbed depends on the Lewis basicity of the medium, and it would be expected that 1ChCl:2glycerol would be less Lewis basic than pure ChCl as the chloride is already hydrogen bonding with the glycerol molecules. To quantify the hygroscopicity, it is helpful to measure the mass increase of both media as a function of humidity and time. Experiments were carried out using thermogravimetric analysis (TGA) with the humidity attachment, and the percentage of the water uptake were calculated at 80 °C throughout the experiment as shown in Fig. 2. The relative humidity was kept at 0% for 60 min, then jumped to 100% and then held constant for 60 min before it went back to 0% humidity and kept at these conditions for another 60 min.

In the first 60 min of the experiment, there is a small decrease in mass which corresponds to the loss of moisture which was originally in the PDES (and to a lesser extent in the gelatin) when the composite was made. During the second period, the water uptake under 100% humidity indicates that the water absorption capacity is reduced after being made into a eutectic mixture, from 30 to 16%. It could be argued that this is obvious since there is less salt in the eutectic mixture, but glycerol is still hygroscopic but less so than ChCl. Furthermore, after removing the humid environment, the ChCl/glycerol manage to quickly return to its original weight within 60 min while the ChCl by itself does not return to its original mass within the time window of the experiment. Given that the patch has a density of 1.25 g cm^−3^, a 1 cm^2^ patch which was 1 mm thick containing 20 wt% PDES would be able to deliver approximately 10 mg of API but withdraw no more than 20 mg of water which should have negligible effect on skin irritation.

### Dissolution studies for pharmaceuticals/PDESs

**Fig. 3:**
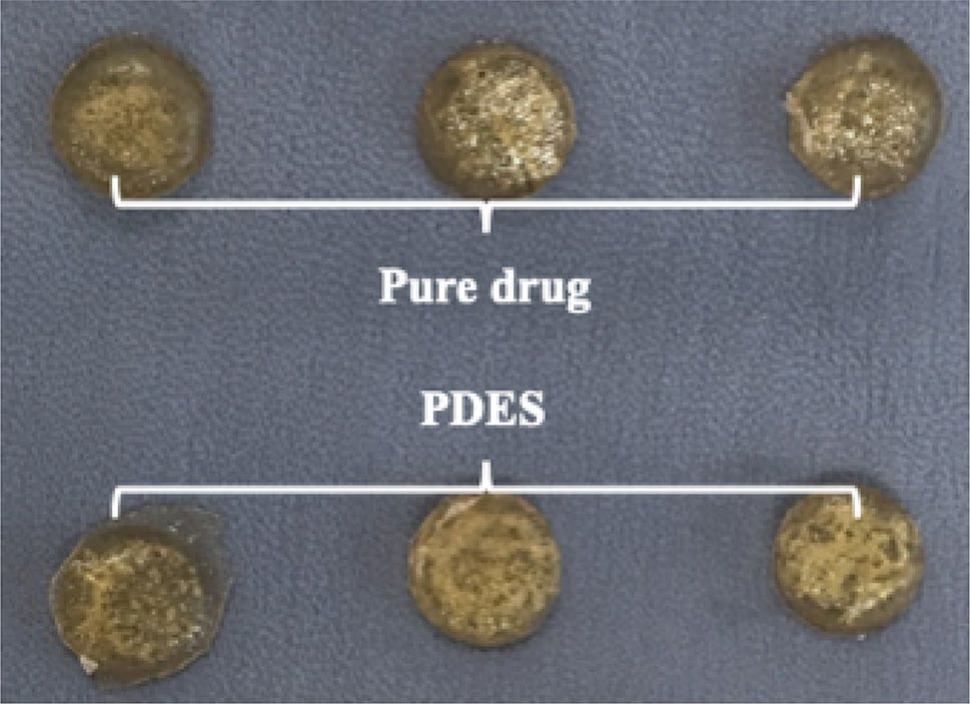
Gelatin-based drug patches on hydrated bovine hide for the transdermal drug delivery test

Dissolution testing is a fundamental part of drug product development and as it is also employed as a quality control tool to monitor batch-to-batch consistency of the drug release from a product. The dissolution curves of different pharmaceuticals and their eutectic formulations examined are shown in Fig. 3S. The release rate profiles were plotted as the percentage of the pure drug in the tablet as a function of time. The dissolution rate of all three PDESs appeared to be faster than the drugs in their pure form. For all three drugs, the concentration of the drug in solution reaches the maxim before the pure drug. Especially for catechol, after 1 s, the dissolution of the eutectic mixture was more than twice that of the pure drug. It should be noted that these experiments were operated with very slow magnetic stirring to avoid further evaporation of the solution, which also lead to the PDESs not being immediately miscible.

The ease of distributing the PDESs into water can be due to that PDESs are liquid which lack of needing to solvate the API from the eutectic system. It has been demonstrated that, by reducing the melting point of a drug, the solubility and transdermal permeation can be increased [19]. Thus, eutectic formation can be used as a method to reduce the melting point of drugs without compromising their pharmaceutical activity. Take for example the imipramine HCl PDES, when a eutectic mixture consisting of a drug with lower solubility and a highly water-soluble carrier, in this case glycerol, was dissolved in an aqueous medium. It could be thought that the carrier would dissolve rapidly, leaving the insoluble drug in an extremely fine state of subdivision. Recent studies by Al-Murshedi et al. have shown that, when DESs are diluted into water, they are not fully miscible but appear to form a bicontinuous microemulsion [20]. This was confirmed by measuring diffusion coefficients of species in mixed water-DES systems and using dynamic light scattering (DLS). The dispersed phase particle size is significantly affected by the relative proportions of DES and water as well as the type of DES. Water can behave as both the dispersed and continuous phases depending on the relative proportions.

**Fig. 4:**
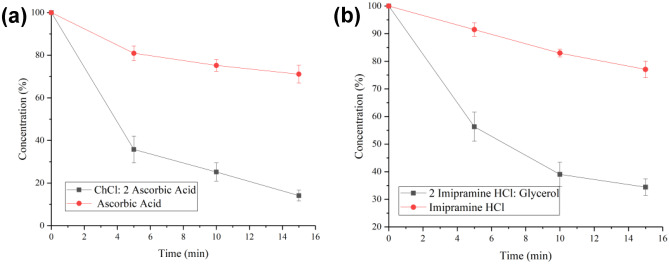
Remaining PDES in patches after different amount of contact time with bovine hide **a** ascorbic acid and ChCl: 2 ascorbic acid, **b** imipramine HCl and imipramine HCl: 2 glycerol. The error bars have been measured for three readings

To determine whether PDESs are totally miscible in water, DLS was carried out, and the results show different behaviour for all three PDESs. Figure 4S shows peaks indicating that ascorbic acid forms clusters which are 100–200 nm in diameter. This is similar to the size of ethaline and reline when dissolved in water. Ascorbic acid does also show a weaker signal for a dispersed phase with a diameter of 0.7–0.8 nm which would correspond to small clusters of 2 or 3 molecules. By contrast, catechol forms clusters which are about 1000 nm in diameter. These are much larger than other DESs tested and may be due to the increased hydrophobicity of catechol. Imipramine hydrochloride has a large signal for clusters with a diameter of c.a. 2 nm which again corresponds to small molecular clusters with probably less than 50 molecules in them There is also another, much weaker, signal for clusters in the range 4000–5000 nm which is probably due to the hydrophobicity of imipramine hydrochloride. A liquid phase dispersed on the nm length scale would be an ideal medium to ensure fast uptake in the body. This result is consistent with results acquired previously where the solubility and dissolution rate of a poorly water-soluble drug can be improved by forming eutectic formulations [21].

### Dissolution studies for protein-based PDES tablets

Gelatin is a traditional water-soluble functional protein of high interest and value. It has the ability of forming transparent gels under specific conditions, which is ideal when it comes to the manufacture of pharmaceutical capsules, ointments, cosmetics, tablet coating and emulsions are concerned [22]. Apart from being a suitable candidate for pharmaceutical use, gelatin was also found that the material property is highly dependent on the procedure of the production, more specifically, it was suspected that the degree of crystallinity in the gelatin is affected by the length of time that the gelatin and PDES mix before they are pressed.

**Fig. 5:**
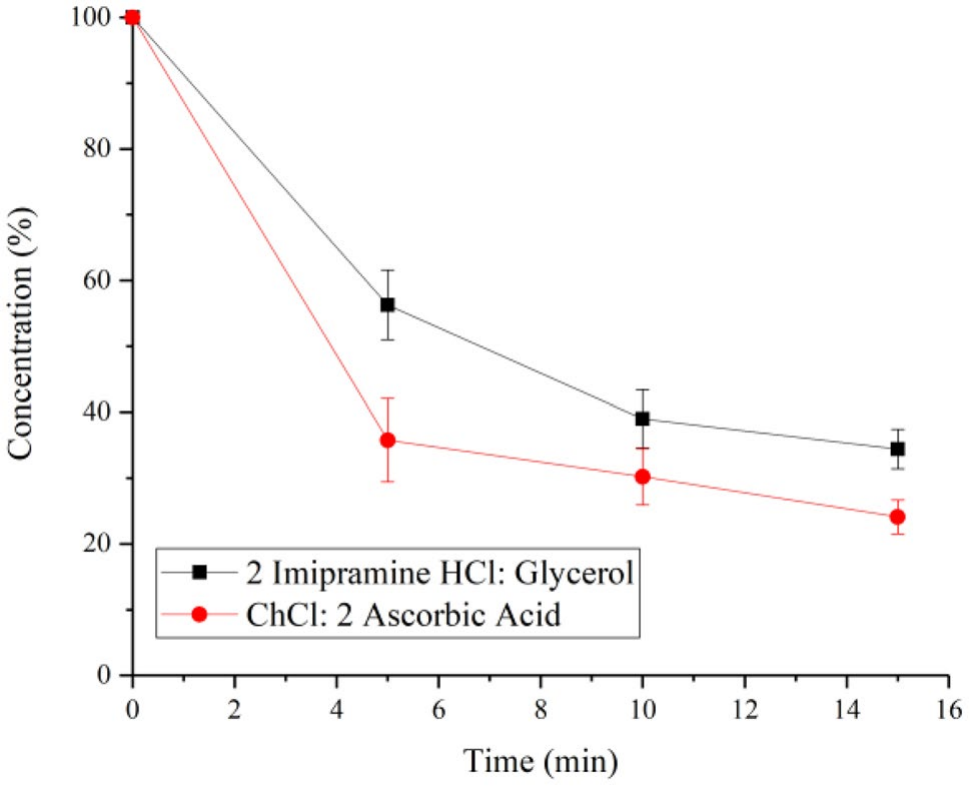
Remaining PDES in patches after different amount of contact time with bovine hide (2ChCl: ascorbic acid and imipramine HCl: 2 glycerol). The error bars have been measured for three readings

To verify this, two different formulation methodologies were tested. Firstly, gelatin was mixed with the PDESs, and the mixture was pressed straight away. In the second the mixtures, gelatin and PDES were mixed and left for 24 h to form a more gel-like texture before it was then pressed into tablets. The tablets produced by these two methods present similar appearance with different feel i.e. the sample which was pressed 24 h after mixing was more flexible and plastic like than that pressed straight away. Here, the amount of the API uptake was estimated based on the concentration increase detected by UV-visible spectrophotometer. Simultaneous dissolution of the gelatin tablet was observed, and they disappeared at the same time that the drug release was completed. It was suggested that the gel structure was destroyed while releasing the API in PDES form. Dissolution study of each drug indicates that the gelatin with different setting time shares the same release profile. However, when it comes to the absolute number of the amount of the drug being released, it can clearly be seen from (a), (b) and (c) in Fig. 5S that the gelatin pill with longer setting time managed to contain more active drug ingredient than the ones pressed right away.

### Transdermal delivery on saline soaked bovine hides

There are numerous methods of simulating drug take up by skin, and one of which is using hydrated leather. Experiments were carried out by placing patches on the hydrated bovine hide sample according to the model presented. Two kinds of drugs were chosen (ascorbic acid: ChCl (1:2) and imipramine HCl: glycerol (1:2)), and each time, two sets of patches (20% wt pure drug in powder form in gelatin and 20% wt PDES formulation in gelatin) pressed after a setting time of 12 h were positioned approximately 3 cm apart (close enough to be in the same area on the leather without being contaminated by each other) as shown in Fig. 3.

In each experiment, two drug patches from each group were removed from the hide piece every 5 min and dissolved in 500 ml of saline solution at 37 °C, with the rotation speed of the paddle adjusted to 100 rpm. The experiment lasted for 15 min, and each experiment was repeated 3 times. After all the patches were fully dissolved, the resultant solutions were then analyzed by direct UV spectrophotometry to determine the remaining drug content. The collected data was calculated and plotted versus time (Fig. 4).

The result in Fig. 4 showed a significant increase in the permeation rate through the leather sample. The percentage of drug release from the PDES patches after 15 min was found to be 65% compared with just imipramine HCl which released only 20%. The result obtained from 2ChCl: ascorbic acid showed a faster release rate than pure ascorbic acid. The reason behind this is because the absorption of the pure drug first requires dissolution in water, which has a finite partition, creating a much more viscous environment.

By comparing imipramine HCl: 2 glycerol and ChCl: 2 ascorbic acid–based PDESs, Fig. 5 confirms this idea where a more viscous solution will have a harder time going across the skin barrier, with 2ChCl: ascorbic acid having only 22% of the API remaining by the end of the experiment.

Research studies undertaken by Abbott et al. found that the DESs have high solubility for polar compounds, which can be deployed to the application of leather processing [23]. It was found that the ingress of species into leather is dominated by interfacial processes, and the use of DES can ensure the species partition into the solid, largely ionic matrix with less loss of the active ingredients. Additionally, the entrapment of the ionic component can act as an in-built plasticiser for the leather. This means that the interaction between the bovine hide and PDESs can also be a contributor for the high speed of the drug delivery. The moisture content of the hydrated leather was determined before and after contact with the gelatin patch, and it was found that there was no noticeable decrease in moisture (26%) confirming the PDESs should result in minimal irritation to the skin.

### Transdermal delivery on porcine cut

An alternative method to test drug delivery is via a porcine cut. Pigs are often considered to be the ideal preclinical model systems thanks to the fact that they have a number of anatomic and physiologic similarities to humans in different systems. This test delivers different results to a bovine hide since it contains no hair, meat and fat whereas a porcine loin cut has the fat and meat content, so will be more hydrophobic.

**Fig. 6:**
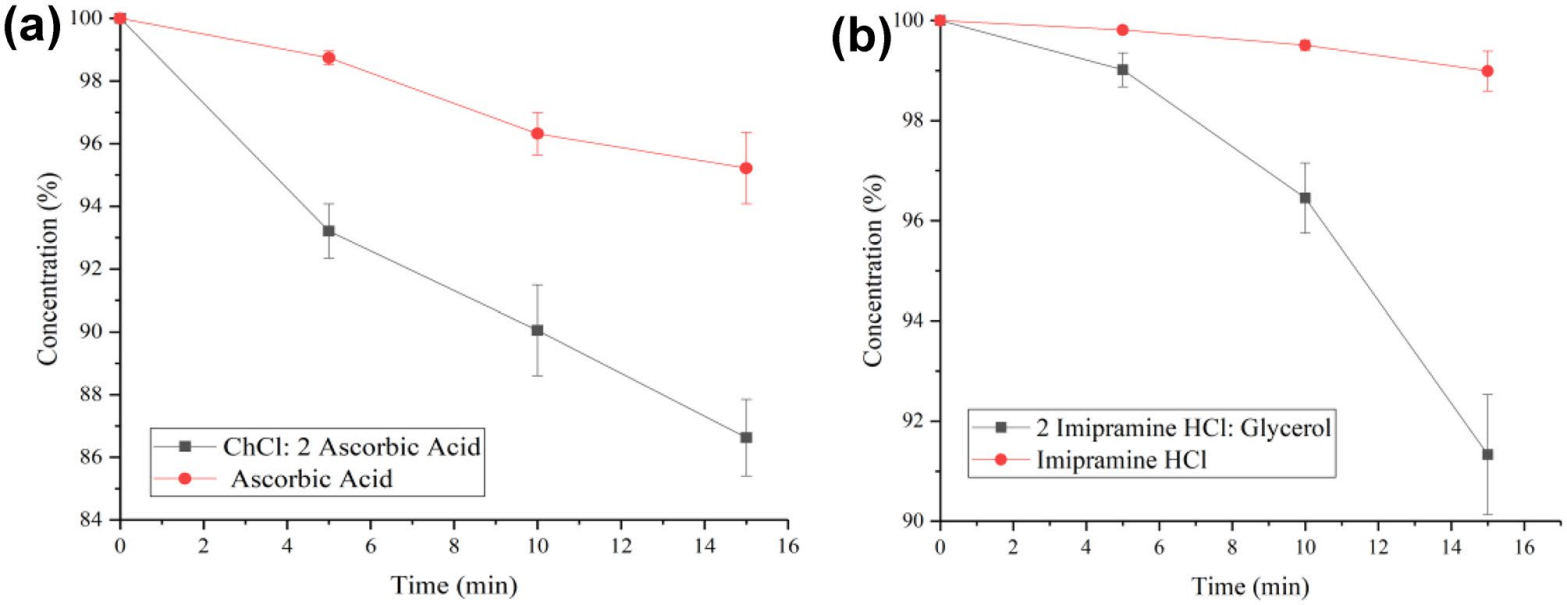
Remaining drug in patches after different amount of contact time with pig skin. **a** Ascorbic acid and 2ChCl: ascorbic acid. **b** Imipramine HCl and imipramine HCl: 2glycerol. The error bars have been measured for three readings

The skin surface of the loin cut was washed with water and soaked in aqueous phosphate buffer of pH 7.4 (Sigma-Aldrich) for about 15 min before it was used for permeation studies. The Hydration level was measured as 27% (comparable to the one obtained from the bovine leather) before the same procedure was carried out as the one on leather as shown in Fig. 6S.

Figure 6 shows a similar enhancing effect of the PDES formulation on the drug delivery behaviour as the one done on bovine hide. However, the delivery speed is much slower for both PDESs. With the ascorbic acid group, only a total of 13% of the drug was released after being formulated into PDES, which is still higher than pure drug by 9%. For the imipramine HCl group, the drug in its original form was barely delivered across the skin, but 2 imipramine HCl: glycerol managed to release 9%, which further confirms the enhancing effect of the eutectic formulations. Both the hydrated leather and porcine cut experiments showed improved uptake of the PDES compared to the pure API although in all cases the hydrated leather showed faster uptake of the active ingredient. This is to be expected and results from the more porous structure of the bovine hide and the hydrophobic nature of the fat layer on the porcine cut coupled with its dense structure.

## Conclusion

This study has shown that three different active pharmaceutical ingredients (APIs), namely, imipramine hydrochloride, ascorbic acid and catechol can be formulated into eutectic mixtures, and the liquids thus formed can be used to plasticise gelatin. The PDES formulations enabled faster dispersion of the API into aqueous solutions, and this enabled the gelatin in pill form to disperse the API faster when dissolved in water. The physical properties of the gelatin PDES blend were found to be relatively insensitive to the type of API used. The water uptake of the PDES is relatively low compared to the constituent components suggesting that the formulations should not increase skin irritation. Two sets of transdermal patch model were tested based on hydrated bovine hide and a porcine loin cut. Both showed significantly faster uptake of two APIs in PDES formulations compared to the pure API. This showed that gelatin-PDESs could be used for both oral and transdermal drug deliveries.

## Supplementary Information

Below is the link to the electronic supplementary material.

Supplementary file1 (DOCX 292 KB)
