# Supplementary material for: Controlled release of pharmaceutical agents using eutectic modified gelatin
Source: Drug Deliv Transl Res. 2021 May 8;12(5):1187–94. doi: 10.1007/s13346-021-00998-3 (PMC8942888; doi:10.1007/s13346-021-00998-3)
Supplement: Supplementary file 1 — Supplementary file1 (DOCX 292 KB) [file 13346_2021_998_MOESM1_ESM.docx]

**Controlled release of pharmaceutical agents using eutectic modified gelatin**

Wanwan Qu^a^ Idrees B. Qader^b^ and Andrew P. Abbott,^a^*

^a^School of Chemistry, University of Leicester, Leicester LE1 7RH, UK

^b^Pharmaceutical Chemistry Department, College of Pharmacy, Hawler Medical University, Erbil, Kurdistan Region, Iraq

**Supplementary information**

| **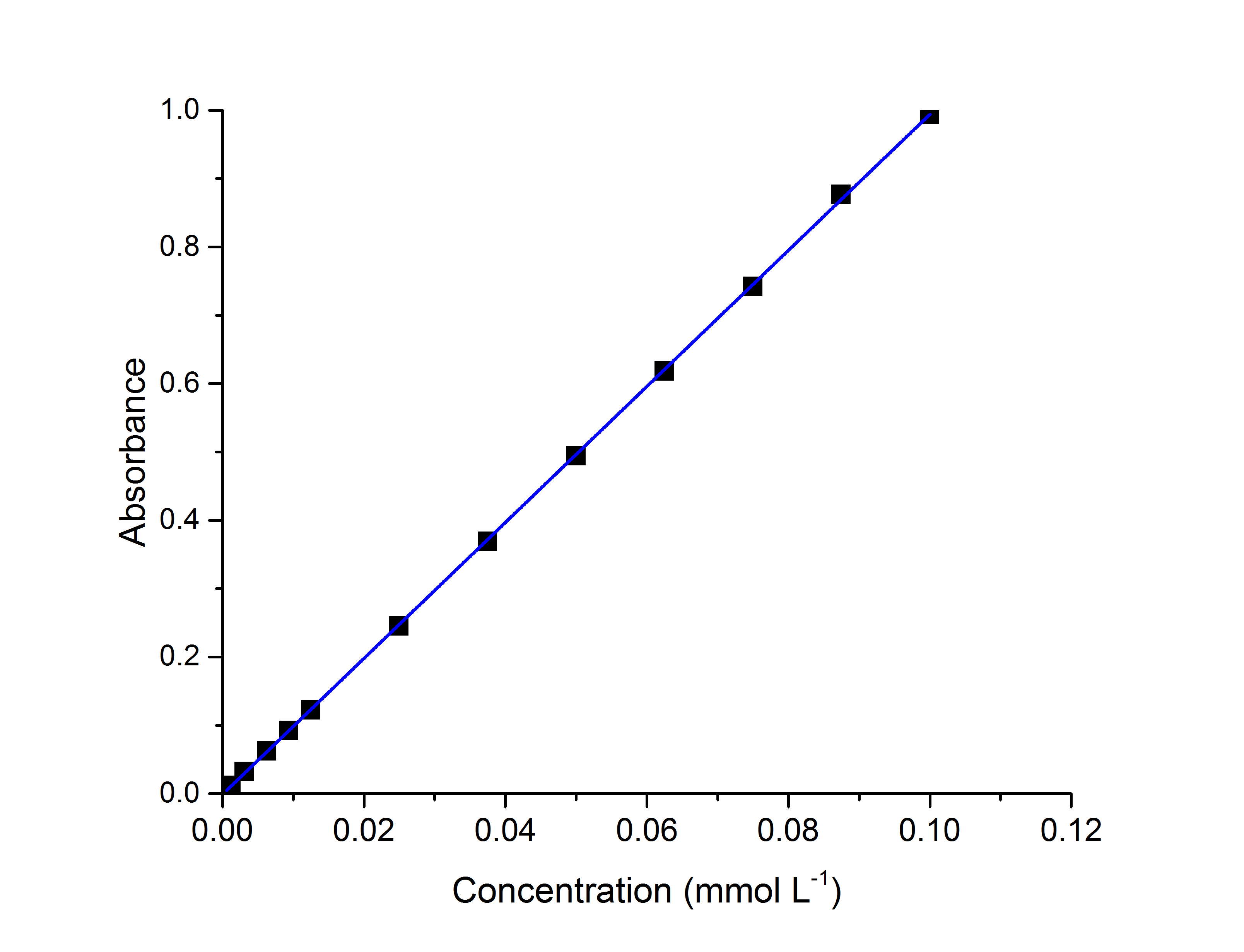**(a) | **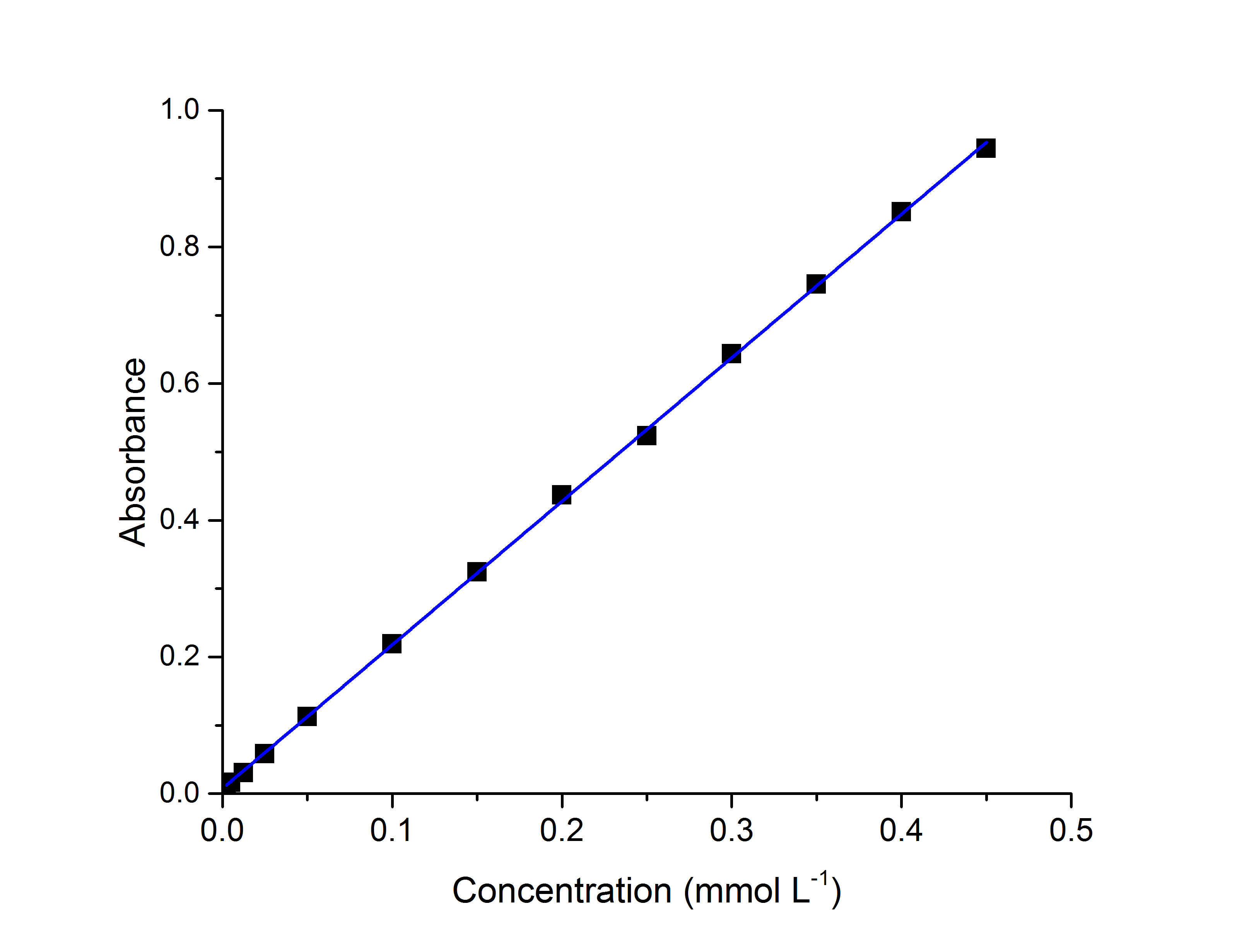**(b) |
| --- | --- |
| **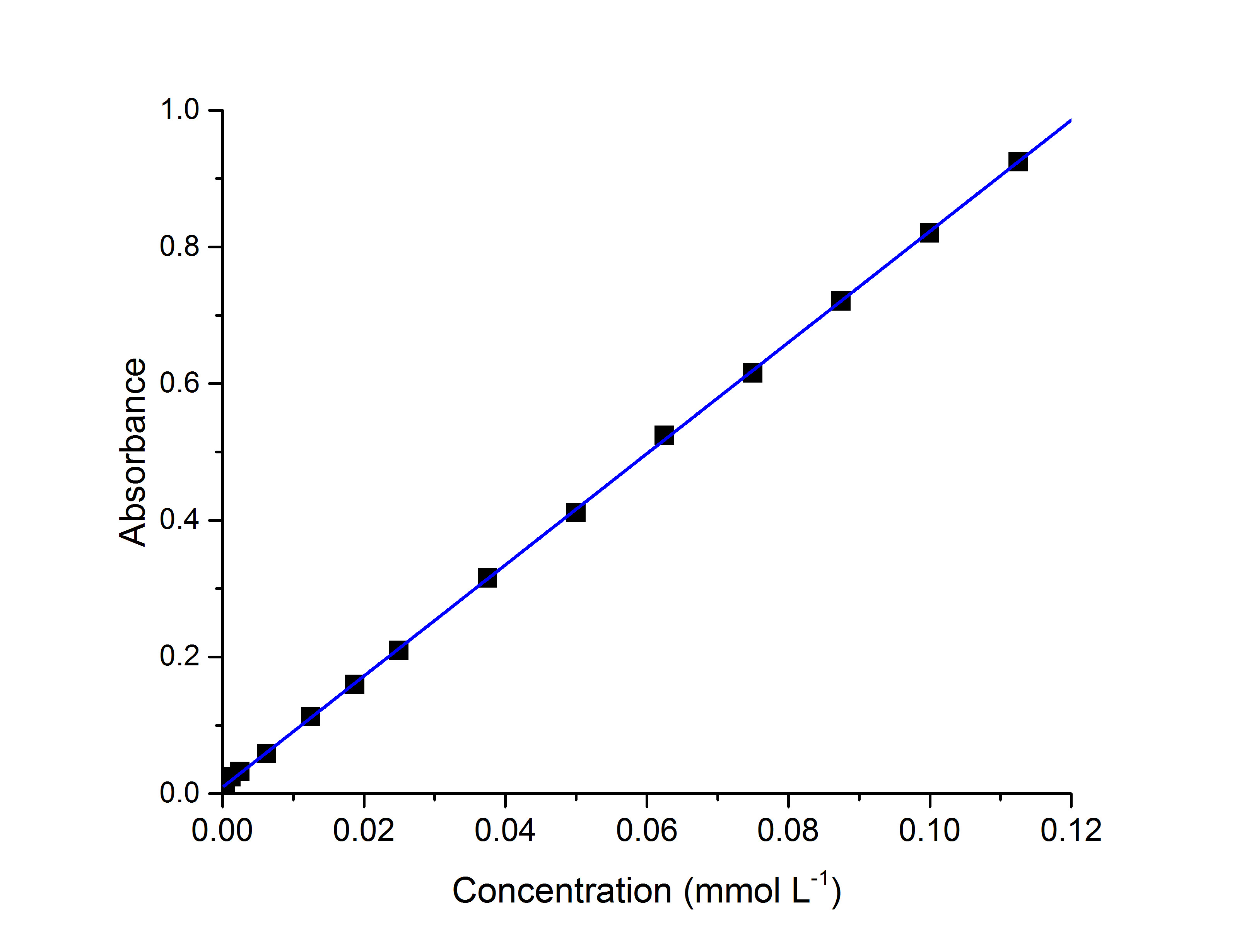**  (c) | |

*Figure 1S: UV-Vis spectroscopy calibration curves for standard concentrations of a) ascorbic acid b) catechol and c) imipramine HCl in 0.1M HCl. The error bars have been measured for three readings.*

| 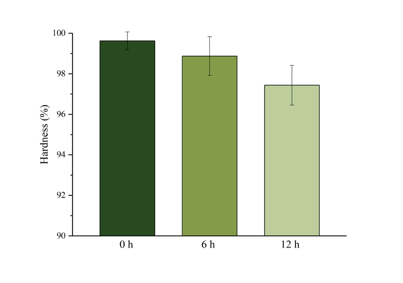 |
| --- |
| ***Figure 2S****: Hardness comparison for gelatin samples plasticised by 20% wt ChCl:2 ascorbic acid after different lengths of setting time. The error bars have been measured for three readings.* |

|   *(a)* |
| --- |
|   *(b)* |
|   *(c)* |

***Figure 3S:*** *Dissolution rate of a) Pure ascorbic acid, ascorbic acid + ChCl, b) Pure catechol, catechol + ChCl and c) Pure imipramine HCl, imipramine HCl + glycerol in 0.1mol /l HCl at 37 ± 0.5°C.*

|  |
| --- |
| (a) |
|  |
| (b) |
|  |
| (c) |

***Figure 4S****: The intensity size distribution of (a) ascorbic acid + ChCl, (b)catechol + ChCl and (c) imipramine hydrochloride + glycerol versus particle diameter in nanometers. The different colors represent repeat measurements of the same sample.*

|   (a): Ascorbic acid + ChCl + gelatin |
| --- |
|   (b): Catechol + ChCl + gelatin |
|   (c): Imipramine HCl + glycerol + gelatin |

***Figure 5S****: dissolution rate of PDES tablets made in two different methods in 0.1mol/l HCl at 37 ± 0.5°C.*

| ***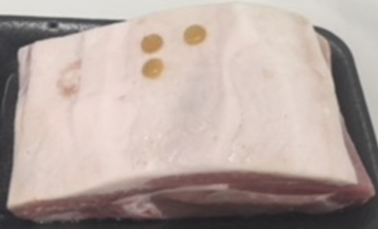*** |
| --- |
| ***Figure 6S****: Gelatin-based* *drug patches on pig loin for transdermal drug delivery test.* |
